# Effect of rice milling, washing, and cooking on reducing pesticide residues

**DOI:** 10.1007/s10068-023-01345-7

**Published:** 2023-06-05

**Authors:** Hyesu Lee, Mihyun Cho, Minsoo Park, Myungheon Kim, Jung-A. Seo, Dong Hyun Kim, Subin Bae, Myeong Seok Kim, Jeong Ah Kim, Joon-Goo Lee, Moo-Hyeog Im

**Affiliations:** 1 Food Additives and Packaging Division, Ministry of Food and Drug Safety, Cheongju, 28159 Republic of Korea; 2 Department of Food Engineering, Daegu University, Gyeongsan, 38453 Republic of Korea; 3 Residues and Contaminants Standard Division, Ministry of Food and Drug Safety, Cheongju, 28159 Republic of Korea; 4 Department of Food Biotechnology, Dong-A University, Busan, 49315 Republic of Korea

**Keywords:** Etofenprox, Flubendiamide, Tebufenozide, Pesticide removal, Processed rice

## Abstract

The effects of milling, washing, and cooking on etofenprox, flubendiamide, and tebufenozide levels in brown and polished rice were investigated by HPLC using a UV detector. The reduction rates of etofenprox, flubendiamide, and tebufenozide after milling were 68.74–93.16%, 64.49–90.25%, and 69.74–92.58%, respectively, 11.64–41.44%, 31.36–65.37%, and 31.61–73.79%, respectively, after washing brown rice, and 30.85–82.08%, 52.13–83.05%, and 43.04–83.89%, respectively, after washing polished rice. The residue levels of the three pesticides in brown rice decreased after electric and pressure cooking by 56.49 and 54.41%, 75.80 and 73.42%, and 70.01 and 71.27%, respectively, and the corresponding levels in polished rice decreased after electric and pressure cooking by 85.58 and 85.82%, 86.70 and 87.06%, and 89.89 and 89.68%, respectively. In conclusion, various processing methods decrease the residual levels of etofenprox, flubendiamide, and tebufenozide in rice.

## Introduction

Pesticides are important agricultural materials for rice production; they prevent and control pests and diseases and improve the quality of agricultural products during cultivation and storage (Kim et al., 2014). Pesticide residues in agricultural products may be unintentionally consumed, leading to chronic toxicity over time; thus, careful pesticide management is a vital undertaking (Hayes and Laws, 1993; Kim et al., 2014; Rhee, 2013). Several countries have established maximum residue limits (MRLs), which are defined as the levels of pesticide residues that will not harm the human body even when consumed every day for a lifetime. The MRLs of the Republic of Korea were established by the Ministry of Food and Drug Safety (MFDS) based on data collected from field trials of pesticide residues in agricultural crops and their measured toxicities (Hayes and Laws, 1993; Lee and Lee, 1997; Park et al., 2005).

Rice is not only the most widely produced and consumed agricultural product in the Republic of Korea but also the most-consumed grain around the world (Kwak et al., 2017). Rice is rich in dietary fiber, fat, proteins, and vitamins, and contains various minerals, including calcium and iron (Choe et al., 2002). The consumption of rice containing various nutrients and functional ingredients can help lower blood sugar and prevent various diseases, such as diabetes, intestinal diseases, arteriosclerosis, and obesity (Ha, 2008). In the Republic of Korea in 2020, the daily average intake of domestic cereals, at 267.23 g/day, was higher than that of vegetables and fruits. Moreover, given that the daily average intake of rice is 121.88 g/day, whereas those of barley and oats are 5.71 and 1.03 g/day, respectively, among the grains produced in the Republic of Korea, rice is the most widely consumed (Korea Health Industry Development Institute, 2022).

Pesticide residues in rice can be removed by milling, washing, and cooking. Different degrees of milling (DOMs) yield different rice products; for brown rice, for example, has a DOM of 0, whereas polished rice has a DOM of 12 (Lee and Eun, 2008). The DOM is a measure of the percentage or degree of bran and germ removed from brown rice kernels. Kim et al. (2014) reported that the residual levels of phenthoate in polished rice decreased by 51% after washing but showed no change after cooking owing to its heat stability. Medina et al. (2020) reported that washing and soaking only reduced pesticide levels by 0.40–4.28%; however, when the rice was presoaked, washed, and cooked, pesticide levels decreased by 57.72, 70.39, and 87.50%, respectively. According to Lee et al. (1997), the reduction rates of organic phosphorus pesticides in rice following milling, washing, and cooking were 50–80%, 51–80%, and 20–99%, respectively. These previous studies clearly demonstrated that the residual amounts of pesticides in rice depend on the method by which it is milled, washed, and cooked.

In the Republic of Korea, MRLs for 167 pesticides have been established for rice, with a residual tolerance level of 0.01–10.0 mg/kg (Pesticides and Veterinary Drugs Information, 2022). Etofenprox is a pyrethroid that inhibits insect neurotransmission (Turner, 2015a, pp. 434–435), flubendiamide is an insecticide that inhibits insect feeding (Turner, 2015b, pp. 496–497), and tebufenozide is an insecticide that acts as a molting hormone (Turner, 2015c, pp. 1057–1058). The MRLs for these pesticides are 1.0, 0.5, and 0.3 mg/kg, respectively. Among the pesticides used for rice, 125 have MRLs of less than 0.2 mg/kg; thus, the MRLs of etofenprox, flubendiamide, and tebufenozide are relatively high (Pesticides and Veterinary Drugs Information, 2022). In the Republic of Korea, MRLs are mainly specified for raw agricultural products, and separate MRLs are defined for some dry foods that exhibit pesticide concentration during processing (Ryu, 2017). As MRLs for brown rice have been established, residual levels of etofenprox, flubendiamide, and tebufenozide can be expected to be significantly reduced during milling, washing, and cooking. However, because research on the changes in the residue levels of these pesticides during rice processing is limited, evaluating realistic exposure to pesticide residues according to actual rice consumption is challenging. If exposure assessment is performed using the MRLs for pesticides, the results may reflect ingested pesticides that have not actually been ingested (Cho, 2022; Lee, 2021; Seo, 2021). Therefore, a study on the changes in the residual levels of etofenprox, flubendiamide, and tebufenozide during rice processing and cooking is essential for realistic pesticide exposure evaluation.

This study was aimed at investigating the effects of various milling, washing, and cooking methods on the levels of etofenprox, flubendiamide, and tebufenozide in brown and polished rice.

## Materials and methods

### Materials

High-purity (> 97.0) etofenprox, flubendiamide, and tebufenozide standards were supplied by Dr. Ehrenstorfer GmbH (Ausgburg, Germany). Stock solutions (1000 mg/L) of these standards were prepared by dissolving them in HPLC-grade acetonitrile (J.T. Baker, Center Valley, PA, USA). HPLC-grade acetonitrile, acetone, and *n*-hexane (all from J.T. Baker) were used for residue extraction and cleanup. Anhydrous sodium sulfate was purchased from Junsei Chemical (Tokyo, Japan). Florisil was purchased from Sigma-Aldrich (St. Louis, MI, USA) and used as an adsorbent for open column chromatography.

### Pesticide application

Etofenprox (10% EC, Bisangtan®, Kyungnong, Seoul, Republic of Korea), flubendiamide (4% SC, Bigany®, HanKookSamgong, Seoul, Republic of Korea), and tebufenozide (8% WP, Mimic®, Kyungnong, Seoul, Republic of Korea) were diluted in water to prepare 0.4 g/L pesticide solutions. The “Samgwang” variety of rice was used, and pesticide-free unhusked rice was purchased. The rice was used in experiments after it had been milled into brown rice. Approximately 20 kg of brown rice was immersed in 40 L of the pesticide solutions for 10 s and then sieved. The brown rice was dried at 30 °C to obtain a moisture content of 15%. The residual amounts of etofenprox, flubendiamide, and tebufenozide in the brown rice after immersion were 31.17, 21.84, and 23.28 mg/kg, respectively.

## Sample processing

### Rice milling

Brown rice was milled to achieve DOMs of 5, 7, 10, and 12 (polished rice) using a rice milling machine (DY-5000R, Dongyangcm, Yeongcheon, Republic of Korea).

### Rice washing

Changes in pesticide residual levels were compared according to the number of times the rice was washed. Briefly, 300 g of rice was added to 450 mL of tap water, swirled by hand approximately 10 times for 15 s, and then drained. These steps were performed between one and five times. Changes in pesticide residue levels were also compared according to the amount of tap water used during rice washing. For this purpose, 300 g of rice was added to 300, 450, and 600 mL of water (i.e., 1, 1.5, and 2 times the weight of rice), swirled by hand approximately 10 times for 15 s, and then drained. The washing step was repeated three times.

### Rice cooking—pressure rice cooker

Husked rice was washed three times with tap water (1.5 times the weight of the rice), soaked in water for 30 min, and then drained. The rice was added to fresh tap water (1.5 times the weight of the rice) and cooked for 40 min using a pressure rice cooker (CRP-HD1010FI, Cuckoo, Siheung, Republic of Korea). Polished rice was processed in the same manner and cooked in the same cooker for the same amount of time.

### Rice cooking—electric rice cooker

Husked rice was washed three times with tap water (1.5 times the weight of the rice), soaked in water for 30 min, and then drained. The rice was added to fresh tap water (1.5 times the weight of rice) and cooked for 40 min using an electric rice cooker (SB-56RC, Kitchen Art, Incheon, Republic of Korea). Polished rice was processed in the same manner and cooked for the same amount of time in the same cooker.

## Sample extraction and cleanup

### Extraction

Approximately 25 g of rice was blended, added to deionized water (30 mL) and the resulting moist sample was allowed to sit for 1 h. Acetonitrile (100 mL) was added to the rice + water mixture, which was subsequently ground and extracted in a high-speed grinder for 3 min at 14,000 rpm. The extract was passed through Celite 545 by suction filtration, and the filtrate was transferred to a 250 mL separatory funnel containing 15 g of NaCl and stirred at 250 rpm for 5 min. The acetonitrile extract was flowed through an anhydrous sodium sulfate layer for dehydration. Subsequently, 40 mL of the extract was collected and dried at 40 ℃ using a rotary evaporator. Finally, the obtained residue was dissolved in 10 mL of methanol:dichloromethane (1:99, v/v).

### Cleanup

An aminopropyl cartridge was activated and conditioned with dichloromethane (5 mL), and 8 mL of the methanol:dichloromethane (1:99, v/v) extract described above was eluted at a speed of 1–2 drops/s and collected. Thereafter, 5 mL of methanol:dichloromethane (1:99, v/v) solvent was eluted and collected to obtain the pesticide residues that might have remained in the cartridge. A nitrogen concentrator was used to concentrate the extracts. The dried samples were redissolved in 4 mL of acetonitrile.

### Instrumental Analysis

The pesticide residues were analyzed using an HPLC system (Shiseido SI2, Tokyo, Japan) coupled with a UV detector (UVD, Shiseido, Tokyo, Japan). The HPLC setup included a SunFire column (length, 250 mm; i.d., 4.6 mm, 5 μm; Waters, Leinster, Ireland) and operated at 40 °C. The injection volume and flow rate were 20 µL and 1.0 mL/min, respectively. UV detection was performed at wavelengths of 225 nm (for etofenprox) and 254 nm (for tebufenozide and flubendiamide). Acetonitrile and deionized water were used as mobile phases A and B, respectively. Gradient elution was performed as follows: 0 min, 40% B; 10–22 min, 57% B; 23–32 min, 95% B; 33–50 min, 100% B, and 51–60 min, 40% B. The retention times of tebufenozide, flubendiamide, and etofenprox were 22.6, 23.2, and 31.9 min, respectively.

### Method validation

The analytical method used to determine pesticide residues in the samples was validated by calculating the linearity, limit of detection (LOD), limit of quantification (LOQ), and recovery according to the manual of the Codex Alimentarius Commission (2019). The linear range of the method was evaluated by calculating regression coefficients (*R*^2^). The LOD and LOQ of each pesticide were estimated by considering signal-to-noise ratios of 3 and 10, respectively, using the background noise from a blank sample for the comparison. Finally, the LOQ was calculated using the formula below. Recovery and repeatability experiments were carried out for determining the accuracy. Triplicates of the rice samples were spiked with the standard solution at three concentration levels: LOQ, 10×LOQ, and 50×LOQ.

$$LOQ\left(\frac{mg}{kg}\right)=\frac{minimum\,detection\,volume\, \left(ng\right)\times final\,solution\,volume\,\left(mL\right)}{injection\,volume\,\left(\mu L\right)\times sample\,weight\,\left(g\right)}\times Dilution\,factor$$

### Statistical analysis

The obtained data were statistically evaluated by one-way analysis of variance using the Statistical Analysis System (version 9.1) software. When significant differences were found (*p* < 0.05), Duncan’s multiple range tests were employed to determine differences among means.

## Results and discussion

### Analytical method validation

The HPLC-UVD method used in this study showed satisfactory performance for the quantification of pesticides in the rice samples. The calibration curves obtained showed good linearity at concentrations of 0.05–5 mg/L, and high *R*^2^ values (> 0.99) were achieved under the given chromatographic conditions. The LODs ranged from 0.012 to 0.015 mg/kg, and the LOQs were between 0.02 and 0.03 mg/kg. For the recovery tests, spiked samples with concentrations equal to the LOQ, 0.5 (approximately 10×LOQ), and 1.25 mg/kg (approximately 50×LOQ) were used. Table 1 shows the recoveries of the pesticides in the rice samples. The recoveries of etofenprox at concentrations of 0.03, 0.5, and 1.25 mg/kg were 90.2–100.1%, 90.4–102.2%, and 92.4–103.7%, respectively. The recovery rates of flubendiamide at concentrations of 0.02, 0.5, and 1.25 mg/kg were 90.2–99.4%, 92.9–103.7%, and 93.2–98.2%, respectively. The recovery rates of tebufenozide at concentrations of 0.02, 0.5, and 1.25 mg/kg were 91.2–103.7%, 99.7–105.6%, and 99.8–107.5%, respectively. The average recoveries of the pesticides ranged from 90.2 to 107.5%. The reproducibility of these measurements was validated by relative standard deviations (RSD) below 8.7% (Table 1). Therefore, the method validation in this study is in accordance with the Codex guidelines, which are internationally accepted guidelines for pesticide analysis (Codex Alimentarius Commission, 2019).

**Table 1:** Recoveries of pesticides in rice samples and processed rice products (*n* = 3)

Pesticide	Sample	Fortification (mg/kg)	Recovery (%) ± SD	RSD	LOQ (mg/kg)
Etofenprox	Brown rice	0.03	93.5 ± 5.0	5.4	0.03
		0.5	102.2 ± 1.1	1.1	
		1.25	103.7 ± 2.4	2.3	
	Polished rice	0.03	100.1 ± 5.8	5.8	
		0.5	90.4 ± 3.3	3.7	
		1.25	98.2 ± 5.0	5.4	
	Cooked brown rice	0.03	90.2 ± 6.1	6.9	
		0.5	96.9 ± 4.6	4.7	
		1.25	96.9 ± 4.5	4.8	
	Cooked polished rice	0.03	97.2 ± 8.5	8.7	
		0.5	99.6 ± 4.0	4.0	
		1.25	92.4 ± 3.3	3.7	
Flubendiamide	Brown rice	0.02	99.4 ± 4.5	4.6	0.02
		0.5	92.9 ± 4.5	4.8	
		1.25	98.2 ± 7.3	7.4	
	Polished rice	0.02	90.7 ± 5.3	5.0	
		0.5	103.7 ± 2.4	2.3	
		1.25	93.2 ± 5.0	5.4	
	Cooked brown rice	0.02	90.2 ± 6.1	6.9	
		0.5	96.9 ± 4.6	4.7	
		1.25	95.1 ± 4.2	4.2	
	Cooked polished rice	0.02	97.2 ± 8.5	8.7	
		0.5	99.8 ± 4.0	4.0	
		1.25	98.2 ± 6.2	4.9	
Tebufenozide	Brown rice	0.02	103.7 ± 4.1	4.0	0.02
		0.5	101.1 ± 0.8	0.8	
		1.25	103.0 ± 4.0	3.9	
	Polished rice	0.02	93.5 ± 1.0	1.1	
		0.5	103.1 ± 3.3	3.2	
		1.25	99.8 ± 4.3	4.4	
	Cooked brown rice	0.02	91.2 ± 6.0	6.6	
		0.5	99.7 ± 6.4	6.5	
		1.25	102.5 ± 4.4	4.3	
	Cooked polished rice	0.02	102.0 ± 8.6	8.4	
		0.5	105.6 ± 1.8	1.7	
		1.25	107.5 ± 7.9	7.3	

*SD* standard deviation

*RSD* relative standard deviation

*LOQ* limit of quantification

## Characteristics of pesticide residues in rice

### Rice milling

Table 2 lists the residual characteristics of pesticides in rice according to the DOM. The etofenprox residual level decreased from 31.17 to 2.13 mg/kg (93.17% decrease) after brown rice was milled into polished rice. Similarly, the flubendiamide and tebufenozide residue levels decreased from 21.84 to 2.13 mg/kg (90.25% decrease) and from 23.28 to 1.73 mg/kg (92.58% decrease), respectively, after brown rice was milled into polished rice. The pesticide residue reduction was greatest in the 12 DOM group. Thus, the more rice is milled, the greater the reduction in pesticide residues. As the three pesticides are nonsystemic pesticides that remain only on the surface of the rice, the reduction rates exceeded 90% after milling. Park et al. (2009) reported azinphos-methyl, chlorpyrifos, chlorpyrifos-methyl, fenitrothion, malathion, and trichlorfon residue reduction rates of 95, 94, 95, 93, 93 and 94%, respectively, after milling wheat. Because these six pesticides are all nonsystemic, they remain on the wheat husk and show reduction rates of over 90% after milling, similar to the results of the present study. Ro et al. (2017) reported that the reduction rates of residual hexaconazole, tricyclazole, and etofenprox were 40–50%, 31.2–35.2%, and 100%, respectively, when brown rice was milled into polished rice. Hexaconazole and tricyclazole are systemic, whereas etofenprox is nonsystemic. The three pesticides in the present study are all nonsystemic; thus, the residue levels of these pesticides are significantly reduced after milling, similar to the results of Ro et al. (2017). The findings thus far indicate that nonsystemic pesticide residue levels can be significantly reduced simply by polishing rice and removing its bran layer.

**Table 2:** Characteristics of pesticide residues in rice according to the degree of milling (*n* = 3)

Processing method	Residual level (mg/kg) Mean ± SD					
	Etofenprox	% Reduction	Flubendiamide	% Reduction	Tebufenozide	% Reduction ^1^
Brown rice	31.17 ± 1.07^a^	–	21.84 ± 0.60^a^	–	23.28 ± 0.40^a^	–
3DOM^2^	9.74 ± 0.35^b^	68.74	7.76 ± 0.54^b^	64.49	7.11 ± 0.52^b^	69.47
5DOM	7.97 ± 0.72^c^	74.41	4.80 ± 0.12^c^	78.01	4.71 ± 0.44^c^	79.77
7DOM	3.54 ± 0.09^d^	88.64	3.32 ± 0.02^d^	84.79	2.86 ± 0.04^d^	87.72
10DOM	2.19 ± 0.09^e^	92.98	2.16 ± 0.11^e^	90.13	1.86 ± 0.11^e^	92.02
Polished rice	2.13 ± 0.05^e^	93.16	2.13 ± 0.07^e^	90.25	1.73 ± 0.04^e^	92.58

^1^((Raw product residue − processing residue)/Raw product residue) × 100

^2^Degree of milling

^a–e^Values followed by the same superscripted letter in the same column are not significantly different (*p* < 0.05)

### Washing—brown rice

Table 3 shows the residual amounts of pesticides in brown rice after washing. The reduction rates of etofenprox were 27.64, 35.28, and 37.73% when the rice was washed using rice:water ratios of 1:1, 1:1.5, and 1:2, respectively. The reduction rates of flubendiamide in rice were 40.50, 46.01, and 47.03%, respectively, when washing using the same rice:water ratios. Similarly, the reduction rates of tebufenozide in rice were 43.62, 49.19, 50.04% after washing using rice:water ratios of 1:1, 1:1.5, and 1:2, respectively. The reduction rates of the three pesticides increased as the amount of water used for washing increased. However, the differences between the pesticide reduction rates of the rice washed using rice:water ratios of 1:1.5 and 1:2 were not significant. Thus, when washing brown rice, washing at rice:water ratio of 1:1.5 is adequate to reduce the pesticide residues. Cho and Im (2022) reported buprofezin reduction rates of 40.5, 41.4, and 46.2% when rice was washed using rice:water ratios of 1:1, 1:1.5, and 1:2, respectively. The authors concluded that the pesticide reduction rate tended to increase as the amount of water for washing increased, similar to the results of the present study.

**Table 3:** Characteristics of pesticide residues in washed brown rice (*n* = 3)

Washing method		Etofenprox		Flubendiamide		Tebufenozide	
		Mean ± SD	% Reduction	Mean ± SD	% Reduction	Mean ± SD	% Reduction
Brown rice		31.17 ± 1.07 ^a^	–	21.84 ± 0.60^a^	–	23.28 ± 0.40^a^	–
Rice: water ratio	1:1	22.55 ± 0.77 ^b^	27.64	13.00 ± 0.22^b^	40.50	13.12 ± 0.04^b^	43.62
	1:1.5	20.17 ± 1.22 ^c^	35.28	11.79 ± 0.29^c^	46.01	11.83 ± 0.21^c^	49.19
	1:2	19.41 ± 0.82 ^c^	37.73	11.57 ± 0.49^c^	47.03	11.63 ± 0.37^c^	50.04
Washing times	1	27.54 ± 1.52 ^b^	11.64	14.99 ± 0.64^b^	31.36	15.92 ± 1.15^b^	31.61
	2	27.41 ± 0.53 ^b^	12.06	12.26 ± 0.06^c^	43.89	11.46 ± 0.54^c^	50.75
	3	21.47 ± 0.73 ^c^	31.12	11.35 ± 0.30^d^	48.02	9.37 ± 0.34^d^	59.75
	4	19.93 ± 0.25 ^d^	36.05	8.65 ± 0.31^e^	60.38	7.96 ± 0.33^e^	65.82
	5	18.25 ± 0.26 ^e^	41.44	7.56 ± 0.16^f^	65.37	6.10 ± 0.54^f^	73.79

^a–f^Values followed by the same superscripted letter in the same column are not significantly different (*p* < 0.05)

The etofenprox reduction rate for brown rice was 11.64–41.44% after 1–5 washes. For flubendiamide, a reduction rate of 31.36–65.37%% was observed after 1–5 washes. Finally, for tebufenozide, a reduction rate in the range of 31.61–73.79% was observed after 1–5 washes. For all three pesticides, the residue reduction rates increased significantly with the number of washes. The octanol–water partition coefficients (*K*_OW_) of etofenprox, flubendiamide, and tebufenozide are 6.9, 4.2, and 4.25, respectively ((Turner, 2015a, b, c). Because the *K*_OW_ of etofenprox indicates that it is more fat-soluble than the two other pesticides, the fact that its washing-induced reduction rate is lower than those of flubendiamide and tebufenozide is expected. Han and Jo (1999) reported that the pesticide residue reduction rates of polished rice treated with captan, carbaryl, chlorpyrifos-methyl, pirimiphos-methyl, fenitrothion, fenthion, and phenthoate were 98, 90, 54.2, 47.5, 50.7, 61.5, and 53.4%, respectively, after five washes. Chlorpyrifos-methyl, pirimiphos-methyl, fenitrothion, fenthion, and phenthoate showed decreases similar to those observed in the present study, whereas captan and carbaryl showed more significant decreases. The *K*_OW_ of captan is 2.8, and its solubility in water is 3.3 mg/L; by comparison, the *K*_OW_ of carbaryl is 1.85, and its solubility in water is 120 mg/L (Turner, 2015d). Pesticides that are fat-soluble, as indicated by a high *K*_OW_ value, show lower reduction rates compared to water-soluble pesticides with low *K*_OW_ values. Thus, the washing-induced reduction rate varies depending on the *K*_OW_ of the pesticides.

### Washing—polished rice

Table 4 shows the residual amounts of the pesticides in polished rice after washing. The etofenprox reduction rates were 66.22, 70.02 and 82.08% when the rice was washed using rice:water ratios of 1:1, 1:1.5, and 1:2, respectively. In the case of flubendiamide, reduction rates of 78.09, 80.08 and 80.69%, respectively, were obtained when the rice was washed using the same rice:water ratios. Finally, tebufenozide reduction rates were 74.21, 76.79 and 78.78%, when the rice was washed using rice:water ratios of 1:1, 1:1.5, and 1:2, respectively. The residual pesticide reduction rates for the polished rice were higher than those for the brown rice after washing using a rice:water ratio of 1:1. Moreover, the etofenprox reduction rate significantly increased as the amount of water increased, but the rates reduction for the two other pesticides did not change significantly with the amount of water used to wash the rice.

**Table 4:** Characteristics of pesticide residues in washed polished rice (*n* = 3)

Washing method		Etofenprox			Flubendiamide		Tebufenozide	
		Mean ± SD	% Reduction		Mean ± SD	% Reduction	Mean ± SD	% Reduction
Polished rice		2.13 ± 0.05^a^		–	2.13 ± 0.07^a^	–	1.73 ± 0.04^a^	–
Rice:water ratio	1:1	0.72 ± 0.02^b^		66.22	0.47 ± 0.01^b^	78.09	0.45 ± 0.01^b^	74.21
	1:1.5	0.64 ± 0.03^c^		70.02	0.42 ± 0.02^b^	80.08	0.40 ± 0.01^bc^	76.79
	1:2	0.38 ± 0.01^d^		82.08	0.41 ± 0.01^b^	80.69	0.37 ± 0.03^c^	78.78
Washing times	1	1.47 ± 0.14^b^		30.85	1.02 ± 0.05^b^	52.13	0.98 ± 0.11^b^	43.04
	2	0.93 ± 0.06^c^		56.12	0.73 ± 0.04^c^	65.57	0.68 ± 0.07^c^	60.78
	3	0.51 ± 0.07^d^		76.08	0.61 ± 0.04^d^	71.44	0.51 ± 0.02^d^	70.31
	4	0.45 ± 0.05^d^		78.80	0.44 ± 0.02^e^	79.26	0.31 ± 0.002^e^	81.87
	5	0.44 ± 0.05^d^		79.15	0.36 ± 0.02^f^	83.05	0.28 ± 0.01^e^	83.89

^a–f^Values followed by the same superscripted letter in the same column are not significantly different (*p* < 0.05)

The etofenprox reduction rate increased from 30.85 to 79.15% when the number of washes increased from one to five. Similarly, the reduction rate of flubendiamide increased from 52.13 to 83.05% and that of tebufenozide increased from 43.04 to 83.89% over the same increase in the number of washes. The reduction rate of the three pesticides tended to increase as number of washes increased. Hwang et al. (2013) compared the reduction rates of two pesticides remaining in polished rice after one, three, five, and seven washes and obtained results similar to those found in the present study, that is, the pesticide reduction rates increased significantly as the number of washes increased. The etofenprox reduction rate was 41.44% for brown rice but 79.15% for polished rice. Although etofenprox is a fat-soluble pesticide, increasing the number of washes for polished rice could increase the pesticide reduction rate. Hwang et al. (2013) reported that for fthalide, which is more fat-soluble than isoprothiolane, the pesticide reduction rate was higher in polished rice than in brown rice, similar to the reduction pattern observed in the present study. Thus, the pesticide residue reduction rate for polished rice can be increased by increasing the number of washes, regardless of the *K*_OW_ value of the pesticide.

### Cooking—brown rice

**Fig. 1:**
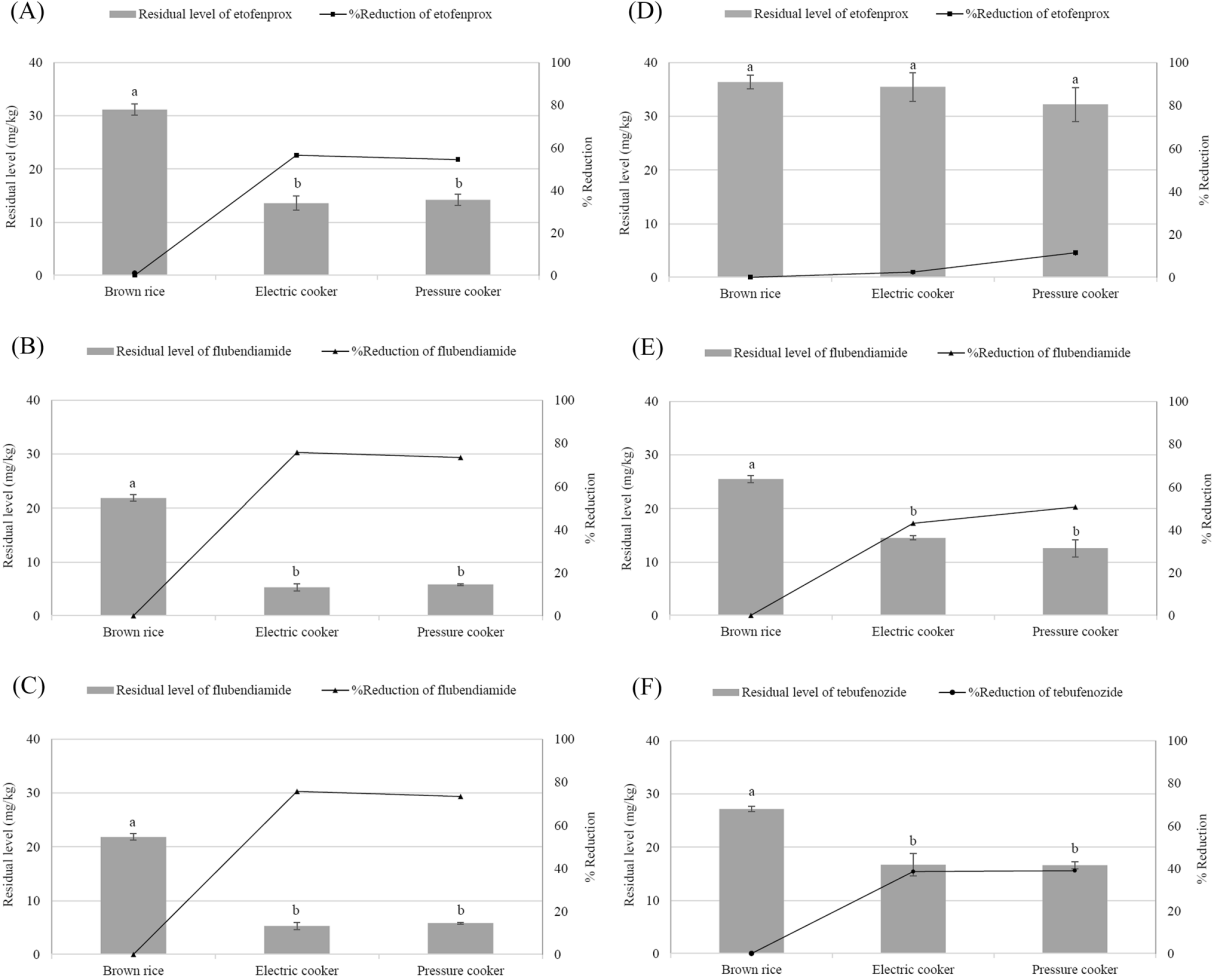
Residual characteristics of (**A**, **D**) etofenprox, (**B**, **E**) flubendiamide, and (**C**, **F**) tebufenozide in cooked brown rice (*n* = 3). (**A**–**C**) wet basis, (**D**–**F**) dry basis. Differences between values marked with the same letter are not statistically significant (*p* < 0.05)

Figure 1 demonstrates the wet and dry basis of the residual levels and reduced rates of the pesticides in brown rice after cooking. On a wet basis, uncooked brown rice contained 31.17 ± 1.07, 21.84 ± 0.60, and 23.3 ± 0.40 mg/kg etofenprox, flubendiamide, and tebufenozide, respectively. The levels of etofenprox, flubendiamide, and tebufenozide in brown rice after electric cooking were reduced by 56.49, 75.80, and 70.01%, respectively. Similar results were obtained after pressure cooking. A dry basis was used to determine the residual amount of pure pesticides, excluding moisture. Uncooked brown rice contained 36.40 ± 1.25, 25.51 ± 0.70, and 27.19 ± 0.47 mg/kg etofenprox, flubendiamide, and tebufenozide, respectively. The levels of etofenprox, flubendiamide, and tebufenozide in brown rice after electric cooking were reduced by 2.47, 43.14, and 38.55%, respectively. Similar results were obtained after pressure cooking. Based on the wet and dry results, the cooking method does not significantly affect the reduction rates for these three pesticides in brown rice. Cho and Im (2022) reported that the reduction rate of buprofezin in brown rice was 51.7 and 55.5% after electric and pressure cooking, respectively. These reduction rates are comparable to those obtained in the present study. The rate of etofenprox reduction was much lower than that of the two other pesticides under both conditions. On a dry basis, there was no significant difference in the residual etofenprox before and after cooking. The vapor pressures of flubendiamide and tebufenozide are < 0.1 and 1.5 × 10^− 4^ mPa (25 °C), respectively, whereas that of etofenprox is 8.13 × 10^− 4^ mPa (25 °C) (Turner, 2015a, b, c). Although the high vapor pressures reflect the volatility of these pesticides, no correlation between vapor pressure and reduction rate was observed in this study.

Hwang et al. (2013) reported that the isoprothiolane and fthalide reduction rates in brown rice were 88.7 and 92.9%, respectively. The vapor pressures of isoprothiolane and fthalide are 4.93 × 10^−1^ and 3 × 10^−3^ mPa (23 °C), respectively (Pesticide Properties DataBase, 2021; Turner, 2015e). Isoprothiolane is significantly more volatile than fthalide, but no significant difference in the reduction rates of these two pesticides was observed. Reports from the Joint Meeting of the FAO Panel of Experts on Pesticide Residues in Food and the Environment and the WHO Core Assessment Group on Pesticide Residues, which were submitted to the Codex Alimentarius Commission to facilitate the establishment of MRLs for pesticides, indicated that the rates of reduction of chlorpyrifos-methyl (Food and Agricultural Organization of the United Nations/World Health Organization [FAO/WHO], 2014), difenoconazole (FAO/WHO, 2015), fenitrothion (FAO/WHO, 2007) in polished rice after cooking were 96–98, 99, and 85–96%, respectively. The vapor pressures of chlorpyrifos-methyl, difenoconazole, and fenitrothion are 3, 3.3 × 10^−5^, and 1.57 mPa (25 ℃), respectively (Turner, 2015f, g, h). In this case, vapor pressure does not appear to have a significant effect on the reduction rates of the three pesticides. Consequently, the lower reduction rate of etofenprox compared with those of the two other pesticides may be attributed to its lower washing-induced reduction rate, as shown in Table 3. After three washes, the reduction rates of etofenprox, flubendiamide, and tebufenozide in brown rice were 31.12, 48.02, and 59.75%, respectively. Hence the rate of reduction due to heating during the cooking process was approximately 10–27%. Residual pesticide levels in brown rice were considerably lowered by washing and heating during the cooking process.

### Cooking—polished rice

**Fig. 2:**
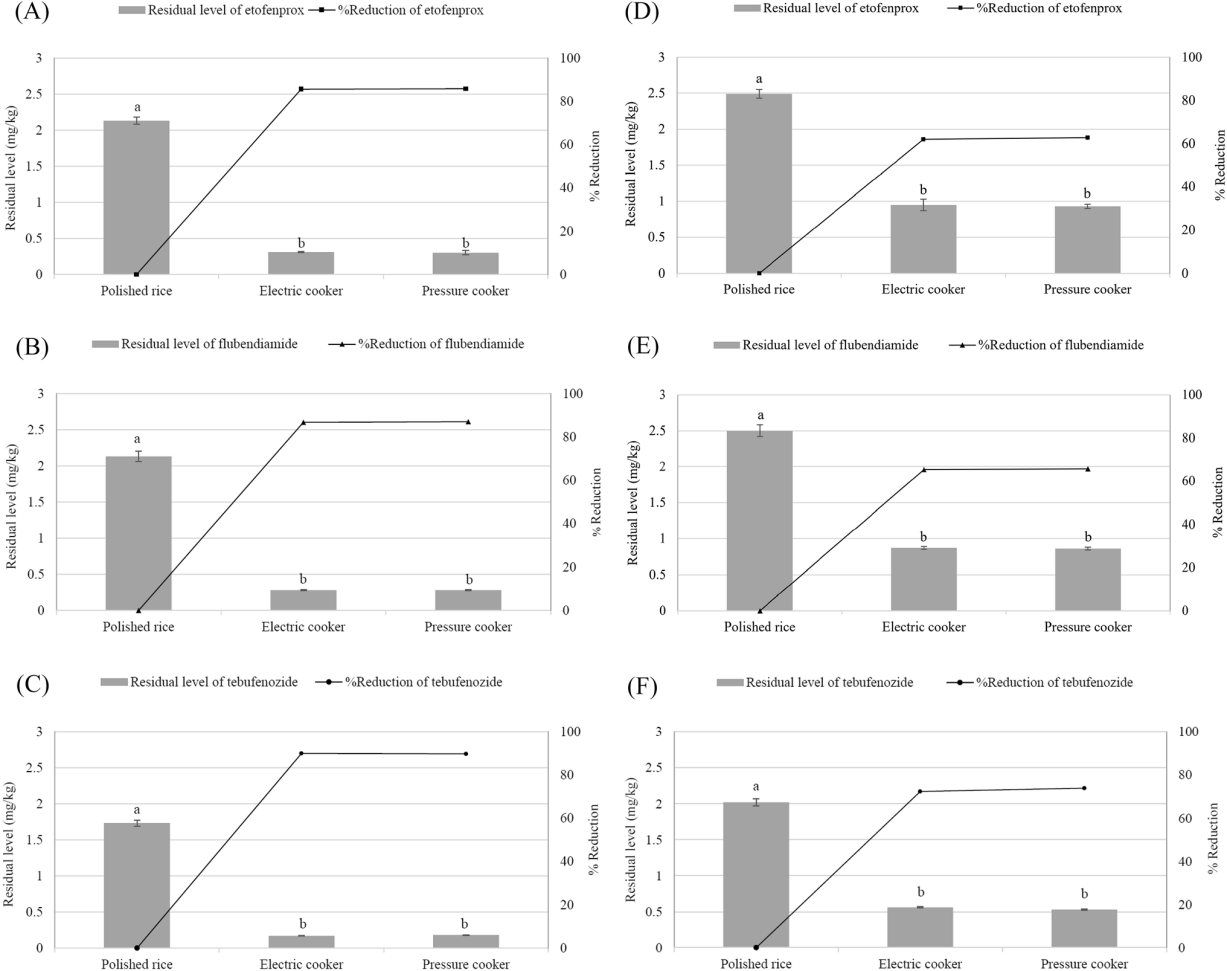
Residual characteristics of (**A**, **D**) etofenprox, (**B**, **E**) flubendiamide, and (**C**, **F**) tebufenozide in cooked polished rice (*n* = 3). (**A**–**C**) wet basis, (**D**–**F**) dry basis. Differences between values marked with the same letter are not statistically significant (*p* < 0.05)

 Figure 2 illustrates the wet and dry basis of the residual levels and pesticide reduction rates of polished rice after cooking. On the wet basis, uncooked polished rice contained 2.13 ± 0.05, 2.13 ± 0.07, and 1.73 ± 0.04 mg/kg etofenprox, flubendiamide, and tebufenozide, respectively. The levels of etofenprox, flubendiamide, and tebufenozide in polished rice after electric cooking were reduced by 85.58, 86.70, and 89.89%, respectively. Similar results were obtained after pressure cooking. On the dry basis, uncooked polished rice contained 2.49 ± 0.06, 2.50 ± 0.08, and 2.02 ± 0.05 mg/kg etofenprox, flubendiamide, and tebufenozide, respectively. The levels of etofenprox, flubendiamide, and tebufenozide in polished rice after electric cooking were reduced by 61.99, 65.31, and 72.34%, respectively. Similar results were obtained after pressure cooking. No significant differences were observed between the reduction rates of the three pesticides under both conditions. Similar to the pesticide reduction rates for brown rice, the effect of vapor pressure was insignificant for polished rice (FAO/WHO, 2007, 2014, 2015; Hwang et al., 2013; Pesticide Properties DataBase, 2021; Turner, [Bibr CR23][Bibr CR31], g, h ).

The reduction rate for etofenprox in brown rice after cooking was lower than the two other pesticides. However, all three pesticides showed similar reduction rates for polished rice after cooking. This observation can be attributed to the similarity in the reduction rates of the three insecticides after washing (Table 4). The reduction rates after three wash cycles were 76.08, 71.44, and 70.31% for etofenprox, flubendiamide, and tebufenozide, respectively. Hence, the reduction rate after cooking was approximately 9–19%. The residual pesticide levels in polished rice were found to be considerably lowered by washing and heating during the cooking process.

In summary, this study investigated the effects of various washing and cooking methods on etofenprox, flubendiamide, and tebufenozide levels in brown and polished rice. The residual pesticide concentrations were decreased by milling, washing, and cooking. In the case of rice milling, the pesticide residues decreased as the DOM increased. As the amount of water relative to rice and the number of washing cycles increased, the residual pesticide concentration decreased. The residual pesticide concentration decreased after the cooking process. The cooking method did not have any significant effect on the residual pesticide concentration. These results presented in this study confirm the reduction of residual pesticide concentrations after processing and provide a realistic guide for pesticide exposure evaluations to enhance public health. These results are not representative of pesticides in general as their intrinsic chemical and biochemical characteristics will dictate their processing requirements.

## References

[CR1] Cho M. Residual characteristics of buprofezin during rice processing and its risk assessment. MS thesis, Daegu University, Republic of Korea (2022)

[CR2] Cho M, Im M-H (2022). Residual characteristics of buprofezin during rice processing. Korean Journal of Food Preservation.

[CR3] Choe JS, Ahn HH, Nam HJ (2002). Comparison of nutritional composition in Korean rices. Journal of the Korean Society of Food Science and Nutrition.

[CR4] CODEX Alimentarius Commission (2019). Procedural Manual.

[CR5] Food and Agricultural Organization of the United Nations/World Health Organization (2007). Pesticide residues in food 2007: Toxicological evaluations. Joint Meeting of the FAO Panel of Experts on Pesticide Residues in Food and the Environment and the WHO Core Assessment Group.

[CR6] Food and Agricultural Organization of the United Nations/World Health Organization. Pesticide residues in food 2015: Report. pp. 547-580. In: Joint Meeting of the FAO Panel of Experts on Pesticide Residues in Food and the Environment and the WHO Core Assessment Group on Pesticide Residues. September 15-24, Geneva, Switzerland. Food and Agricultural Organization of the United Nations/World Health Organization, Rome (2015)

[CR7] Food and Agricultural Organization of the United Nations/World Health Organization. Pesticide residues in food 2013: Report. pp 357-360. In: Joint Meeting of the FAO Panel of Experts on Pesticide Residues in Food and the Environment and the WHO Core Assessment Group on Pesticide Residues. September 17-26, Geneva, Switzerland. Food and Agricultural Organization of the United Nations/World Health Organization, Rome (2014)

[CR8] Ha TY (2008). Health functional properties of rice. Food Industry and Nutrition.

[CR9] Han SH, Jo HB (1999). Effect of storage temperature, washing, and cooking on postharvest-treated pesticide residues in polished rice. Journal of Food Hygiene and Safety.

[CR10] Hayes WJ, Laws ER (1991). Handbook of Pesticide Toxicology.

[CR11] Hwang LH, Kim AK, Jung BK, Lee JK, Shin JM, Park YH, Park HW, Kim MJ, Park KA, Yun ES, Kim MS (2013). Removal of pesticides during washing and cooking of rice. Journal of Food Hygiene and Safety.

[CR12] Kim NH, Lee MG, Lee SR (1996). Elimination of phenthoate residues in the washing and cooking of polished rice. Korean Journal of Food Science and Technology.

[CR13] Kim JY, Lee SM, Lee HJ, Chang MI, Kang NS, Kim NS, Kim KJ, Cho YJ, Jeong JY, Kim MK, Rhee GS (2014). Monitoring and risk assessment of pesticide residues for circulated agricultural commodities in Korea-2013. Journal of Applied Biological Chemistry.

[CR14] Korean Health Industry Development Institute. Food intake. Available from: https://www.khidi.or.kr/kps/dhraStat/result2?menuId=MENU01653&gubun=&year=2020. Accessed Nov. 15, 2022.

[CR15] Kwak JE, Yoon MR, Lee JS, Lee JH, Ko SH, Tai T, Won YJ (2017). Morphological and starch characteristics of the Japonica rice mutant variety Seolgaeng for dry-milled flour. Food Science and Biotechnology.

[CR16] Lee H-S. Residual characteristics of etofenprox during processing of rice food. MS thesis, Daegu University, Republic of Korea (2021)

[CR17] Lee BD, Eun JB (2008). Rice processing in food industry. Food Industry and Nutrition.

[CR18] Lee MG, Lee SR (1997). Reduction factors and risk assessment of organophosphorus pesticide in Korean foods. Korean Journal of Food Science and Technology.

[CR19] Medina MB, Munitz MS, Resnil SL (2020). Effect of household rice cooking on pesticide residues. Food Chemistry.

[CR20] Park KS, Im MH, Choi DM, Jeong JY, Chang MI, Kwon KI, Hong MK, Lee CW (2005). Establishment of Korean maximum residue limits for pesticides in foods. Korean Journal of Pesticide Science.

[CR21] Park SY, Park KS, Im MH, Choi H, Chang MI, Kwon CH, Kim SG, Lee HK, Hong MK, Shim JH, Kim JH (2009). Studies for the processing factors of pesticides during the milling of wheat grain. Korean Journal of Pesticide Science.

[CR22] Pesticide Properties DataBase. Properties on phthalide. http://sitem.herts.ac.uk/aeru/ppdb/en/Reports/1212.htm. Accessed Nov. 15, 2021

[CR23] Pesticides and Veterinary Drugs Information. MRL set on rice. Available from: https://residue.foodsafetykorea.go.kr/. Accessed Oct. 26, 2022

[CR34] Rhee GS. Monitoring of Pesticide Residues in Agricultural Products - 2013. Ministry of Food and Drug Safety, Cheongju, Republic of Korea (2013)

[CR24] Ro JH, Lim SJ, Jin YD, Kim DB, Choi GH, Kim SS, Lee HW, Park JH (2017). Residue patterns of hexaconazole, tricyclazole, etofenprox and imidacloprid in polished and unpolished rice. Korean Journal of Pesticide Science.

[CR60] Ryu YG. The principle of setting standards for food, etc. Ministry of Food and Drug Safety, Cheongju, Republic of Korea. pp. 12-33 (2017) https://www.mfds.go.kr/brd/m_218/view.do?seq=30185&srchFr=&srchTo=&srchWord=%EA%B8%B0%EC%A4%80+%EC%84%A4%EC%A0%95+%EC%9B%90%EC%B9%99&srchTp=0&itm_seq_1=0&itm_seq_2=0&multi_itm_seq=0&company_cd=&company_nm=&Data_stts_gubun=C9999&page=1

[CR25] Seo J-A. Residual characteristics of flubendiamide and etofenprox during rice washing and cooking process. MS thesis, Daegu University, Republic of Korea (2021)

[CR26] Turner JA (2015). The Pesticide Manual: A World Compendium.

[CR27] Turner JA (2015). The Pesticide Manual: A World Compendium.

[CR28] Turner JA (2015). The Pesticide Manual: A World Compendium.

[CR29] Turner JA (2015). The Pesticide Manual: A World Compendium.

[CR30] Turner JA (2015). The Pesticide Manual: A World Compendium.

[CR31] Turner JA (2015). The Pesticide Manual: A World Compendium.

[CR32] Turner JA (2015). The Pesticide Manual: A World Compendium.

[CR33] Turner JA (2015). The Pesticide Manual: A World Compendium.

